# Effect of PM_2.5_ Levels on Respiratory Pediatric ED Visits in a Semi-Urban Greek Peninsula

**DOI:** 10.3390/ijerph18126384

**Published:** 2021-06-12

**Authors:** Nikolaos Kanellopoulos, Ioannis Pantazopoulos, Maria Mermiri, Georgios Mavrovounis, Georgios Kalantzis, Georgios Saharidis, Konstantinos Gourgoulianis

**Affiliations:** 1 Department of Respiratory Medicine, Faculty of Medicine, University of Thessaly, BIOPOLIS, 41110 Larissa, Greece; ndkanell@yahoo.com (N.K.); pantazopoulosioannis@yahoo.com (I.P.); kgourg@uth.gr (K.G.); 2 Department of Emergency Medicine, Faculty of Medicine, University of Thessaly, BIOPOLIS, 41110 Larissa, Greece; gmavrovounis@gmail.com; 3 Department of Anesthesiology, Faculty of Medicine, University of Thessaly, BIOPOLIS, 41110 Larissa, Greece; 4 Department of Mechanical Engineering, University of Thessaly, Leoforos Athinon, 8 Pedion Areos, 38334 Volos, Greece; george.kalantzis4@gmail.com (G.K.); saharidis@gmail.com (G.S.)

**Keywords:** PM_2.5_, air pollution, respiratory diseases, asthma, pneumonia, upper respiratory infections, emergency department

## Abstract

Ambient air pollution accounts for an estimated 4.2 million deaths worldwide. Particulate matter (PM)_2.5_ particles are believed to be the most harmful, as when inhaled they can penetrate deep into the lungs. The aim of this study was to examine the relationship between PM_2.5_ daily air concentrations and pediatric emergency department (ED) visits for respiratory diseases in a Greek suburban area. All pediatric ED visits for asthma-, pneumonia- and upper respiratory infection (URI)-related complaints were recorded during the one-year period. The 24-h PM_2.5_ air pollution data were prospectively collected from twelve fully automated air quality monitoring stations. The mean annual concentration of PM_2.5_ was 30.03 μg/m^3^ (World Health Organization (WHO) Air Quality Guidelines (AQG) Annual mean concentration: 10 μg/m^3^). PM_2.5_ levels rose above the WHO Air Quality Guidelines (AQG) 24-h concentrations (25 μg/m^3^)), 178 times (48.6% of the study period). When PM_2.5_ levels were above the daily limit, an increase of 32.44% (*p* < 0.001) was observed in daily pediatric ED visits for respiratory diseases and the increase was much higher during spring (21.19%, *p* = 0.018). A 32% (*p* < 0.001) increase was observed in URI-related visits, when PM_2.5_ levels were ≥25 μg/m^3^, compared to the mean daily visits when PM_2.5_ levels were <25 μg/m^3^. Air pollution levels were associated with increased pediatric ED visits for respiratory-related diseases.

## 1. Introduction

Air pollution and its detrimental effects on human health have become a prevalent worldwide problem, causing approximately 4.2 million premature deaths each year [1]. Specifically, fine particulate matter with an aerodynamic diameter of equal or less than 2.5 μm (PM_2.5_) is a predominant anthropogenic air pollutant produced mainly through the combustion of wood and fuel [2] and has been ranked as the 4th leading risk factor for disease in China [3]. World Health Organization’s (WHO) air quality guidelines propose that PM_2.5_ exposure levels should not exceed a daily limit of 25 μg/m^3^ [4], however, people living in several urban and suburban areas worldwide are regularly exposed to higher levels [5,6].

The harmful effects of short-term and long-term exposure to PM_2.5_ have been previously studied and corroborated by several studies [7,8,9]. Exposure to various PM_2.5_ components has been shown to be a significant risk factor for both cardiovascular and respiratory diseases [8]. High levels of PM_2.5_ have been strongly associated with the development of severe chronic respiratory diseases [10], as well as increased respiratory-related mortality [7]. Moreover, high levels of daily PM_2.5_ exposure are associated with increased Emergency Department (ED) visits for respiratory symptoms in both adult and pediatric populations [11,12]. Specifically, harmful levels of air pollution have been correlated with an increased number of pediatric ED visits for asthma-like symptoms [12,13], as well as pneumonia and bronchitis [12].

The aim of this study was to examine the relationship between PM_2.5_ daily air concentrations and pediatric ED visits for respiratory diseases in a Greek suburban area where significant air pollution has been previously reported [14].

## 2. Materials and Methods

### 2.1. Study Design and Population

The current project constitutes a retrospective analysis of prospectively collected data. The population of the study was defined as all the patients who visited the pediatric ED of the General Hospital of Volos between 1 March 2019 and 29 February 2020 for asthma, pneumonia and upper respiratory infection (URI)-related complaints. The study protocol was approved by the scientific board of our institution (2/7-2-2019). The electronic database system and the paper records of the Pediatric ED of our hospital were used to identify and collect patients’ data on a daily basis. The local medical school collected, organized and analyzed the data collected.

The following inclusion and exclusion criteria were used to select the patients that were included in the analysis: (1) residents of Volos city, (2) presenting in the pediatric ED with (3) respiratory-related conditions (URI, pneumonia, asthma). 

The study was conducted according to the “Declaration of Helsinki” [15] and the “Health Insurance Portability and Accountability Act of 1996” (HIPAA) [16].

### 2.2. Patient Data

The following patient data were collected: date of visit, age, gender, address, main complaint and diagnosis.

### 2.3. Network Implementation and PM_2.5_ Levels Measurements

The daily 24-h PM_2.5_ air pollution data were prospectively collected from twelve fully automated air quality monitoring stations located in the center and the greater area of Volos, during the study period. The fully automated air quality monitoring network was established by the GreenYourAir research team. 

The GreenYourAir monitoring network consists of twelve measuring devices (GreenYourAir Device 1178/PM_2.5_). The light-scattering method was utilized for the data collection, as previously described [17,18]. The main parts of the device are a sensor that provides data for the concertation of PM_2.5_, temperature and relative humidity, an I/O expansion shield and an Arduino YUN rev. 2. The programming language of the device is C++. The devices collected data every second and were working 24 h per day. 

For the creation of the network, the GreenYourAir research team developed a mathematical formula and an optimization model to determine the locations of the twelve sensors. The city of Volos was divided into five zones (commercial and recreational zone, high-density residential zone, medium-density residential zone, low-density residential zone and industrial zone). The traffic jam of the city was divided into three types (high traffic jam, medium traffic jam and low traffic jam). In the city of Volos there is a commercial port, a passenger port, an intercity bus station, an urban bus station and a train station. The city’s main sources of heating are oil, natural gas and fireplaces. Outside the city to the west, about 6 km from the center, there is the first industrial zone of Volos. Outside the city to the east, about 3.5 km from the center of the city, there is the Lafarge Cement Volos Plant and the ELINOIL Petroleum company. The geographical location of the city and the geomorphological characteristics were also analyzed. A mathematical formula was developed by the research team to separate the city into smaller areas and determine the number of sensors per area.

**Figure 1:**
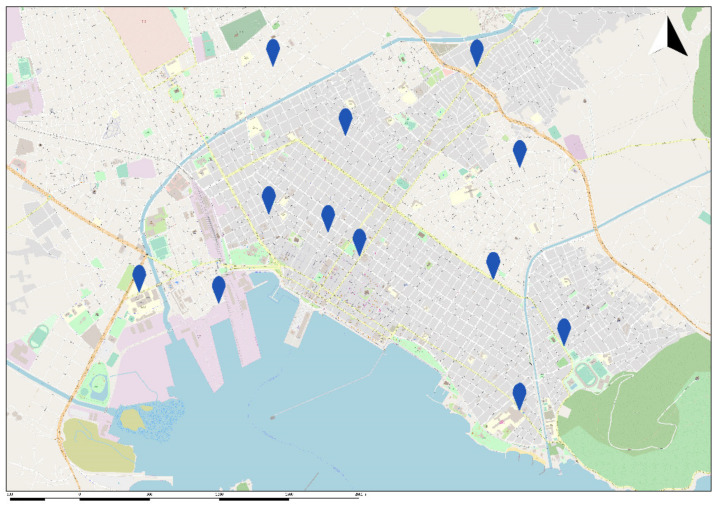
Map of the city of Volos and the location of PM_2.5_ sensors. Markers are pointing at the location of PM_2.5_ sensors.

To determine the number of sensors per area the research team analyzed the specific characteristics of each area such as parks, main roads, sport facilities, schools, universities, sources of heating, traffic jam, type of zones, etc. To determine the locations of the sensors and the number of sensors per area, the research team developed an optimization model taking into consideration the number of sensors per area, the distance between the sensors in the same area, the distance between the sensors of different areas and the specific characteristics of each area. A map of the city of Volos and the location of sensors is presented in Figure 1.

The GreenYourAir team developed a calibration methodology to validate the accuracy of the GreenYourAir Device 1178/PM_2.5_. The calibration methodology was developed in two phases. At the first phase of development and testing, all the sensors were tested under various laboratory conditions with reference equipment. At the second phase of development and testing, the reference equipment installed in each sensor’s location (reference to map?) (Figure 1) and the same procedure took place with both sensors working in real conditions. With the data of the reference equipment, the temperature and the humidity the calibration method developed and the accuracy of the values of PM_2.5_ of the GreenYourAir Device 1178/PM_2.5_ validated. The reference equipment follows EU standards EN 14907:2005. The reference equipment is a gravimetric device with filters that collects the PM_2.5_ and the standard procedure of the manufacture followed to export the values of PM_2.5_.

GreenYourAir network works 24/7 from 1 March 2019 until today. Information about the levels of PM_2.5_ in real-time of the city of Volos is available at http://greenyourair.org/ (accessed on 9 June 2021).

### 2.4. Statistical Analysis

Statistical analysis was performed using the SPSS statistical package (IBM Corp. Released 2016. IBM SPSS Statistics for Windows, Version 24.0. Armonk, NY, USA; IBM Corp). Between groups, comparisons for continuous variables were performed using independent samples *t*-test and analysis of variance (ANOVA), as appropriate. Regression analysis was performed to describe the correlation between the PM_2.5_ levels and the number of ED visits. All significance tests were two-tailed and *p* values < 0.05 were considered to be significant. 

## 3. Results

### 3.1. Data Collection and Patient Characteristics

For the 12-month period between March 2019 and February 2020, the “GreenYourAir” research team collected data concerning the concentration of PM_2.5_ in the atmosphere of the city of Volos. Concurrently and in collaboration with the local medical school and the General Hospital of Volos, a total of 4271 respiratory tract-related visits to the pediatric ED were recorded. Supplementary Table S1 summarizes the ages of the patients who were included in our study during the study period. 

There were 208 asthma-related [Mean age (Standard Deviation): 6.8 (4.18); Male gender: 54.8%], 93 pneumonia-related [Mean age (Standard Deviation): 7.72 (4.86); Male gender: 39.8%] and 3924 acute URI-related [Mean age (Standard Deviation): 6.28 (4.52); Male gender: 52.4%] ED visits. 

### 3.2. ED Visits and PM_2.5_ Levels

**Figure 2:**
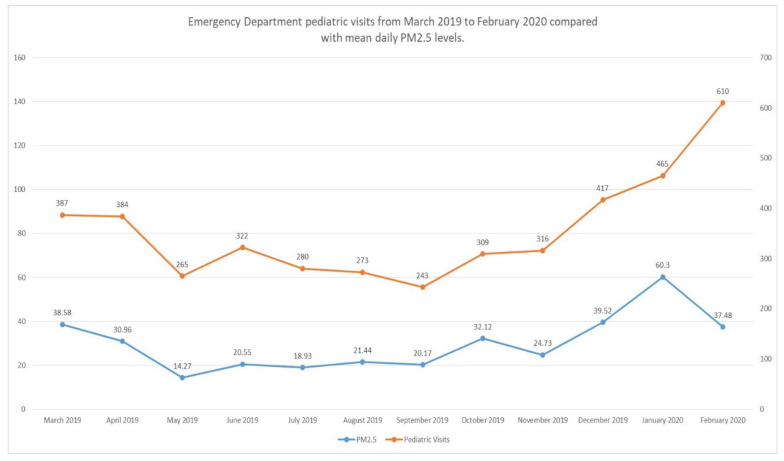
Emergency Department pediatric visits from March 2019 to February 2020 compared with mean daily PM_2.5_ levels.

Figure 2 depicts the number of ED visits per month between March 2019 and February 2020 along with the corresponding PM_2.5_ levels. PM_2.5_ levels were found to be higher in winter when compared with spring (mean difference: 17.71, *p*: <0.001), summer (mean difference: 25.64, *p*: <0.001) and autumn (mean difference: 20.20, *p*: <0.001).

The mean annual concentration of PM_2.5_ in the city of Volos was 30.03 (17.47) μg/m^3^ (WHO yearly limit: 10 μg/m^3^). As presented in Table 1, PM_2.5_ levels rose above 25 μg/m^3^, the daily limit suggested by WHO, 178 times (48.6% of the study period). When PM_2.5_ levels were above the daily limit, an increase of 32.44% (13.35 vs. 10.08 visits/day; *p*: <0.001) was observed in daily pediatric ED visits for respiratory diseases. Table 1 presents the results for each individual disease category. Moreover, a statistically significant difference in pediatric ED visits of the next day was noted when PM_2.5_ levels of the previous day raised above 25 μg/m^3^ (*p* < 0.001). When further analysis was performed based on the season (Table 2), it became apparent that the increase in ED visits was much higher during winter (14.20%) and spring (21.19%). 

It is interesting to note that, despite the lower levels of PM_2.5_ during February compared to January as depicted in Figure 1, the number of ED visits was higher than the previous month. The first weeks of February coincided with a severe rise in influenza outbreaks throughout Europe [19]. It is possible that the rise in pediatric ED visits for respiratory symptoms could be attributed to a local outbreak of influenza or another viral pathogen infecting the respiratory system.

### 3.3. Association of Upper Respiratory Tract Infection, Asthma and Pneumonia with PM_2.5_ Levels

Regarding URI-related visits, a statistically significant increase was observed when PM_2.5_ levels were ≥25 μg/m^3^, compared to the mean daily visits when PM_2.5_ levels were <25 μg/m^3^ (9.28 vs. 12.25 visits/day; *p*: <0.001) (Table 1); the increase was statistically significant during spring (Table 3). The number of URI-related visits was significantly higher in winter when compared with spring (mean difference: 4.59, *p*: <0.001), summer (mean difference: 5.94, *p*: <0.001) and autumn (mean difference: 6.30, *p*: <0.001).

Regarding asthma and pneumonia patients, no statistically significant difference was found when PM_2.5_ levels were ≥25 μg/m^3^, compared to the mean daily visits when PM_2.5_ levels were <25 μg/m^3^ (Table 1).

**Figure 3:**
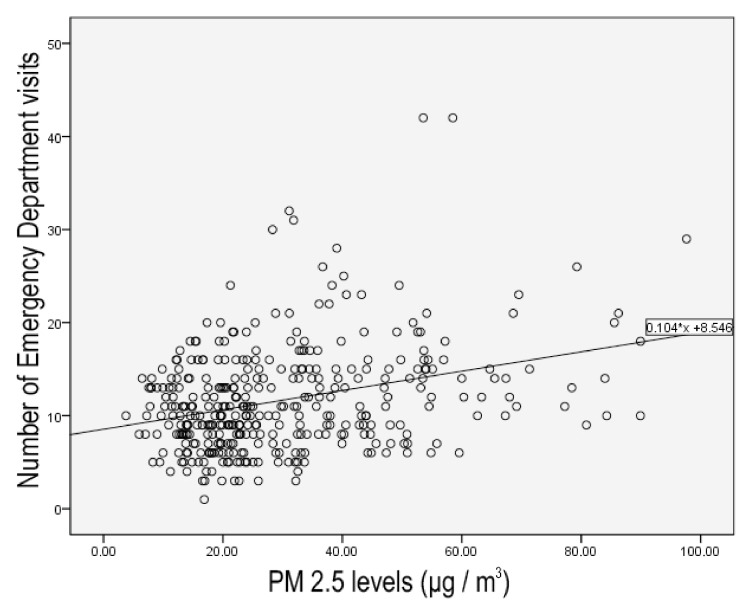
Regression analysis between ED Visits and PM_2.5_ levels.

Using regression analysis, we identified that PM_2.5_ levels were linearly correlated (*p* = 0.000) (r Square = 0.103) with the total number of ED visits. This correlation is described by the following equation: total number of ED visits = 8.546 + 0.104 × PM_2.5_ levels (Figure 3).

## 4. Discussion

Our study demonstrated that high PM_2.5_ levels were associated with an increase in pediatric ED visits for pulmonary diseases. Specifically, we estimated that there was an increase of pediatric ED visits for URI (32%), asthma exacerbations (19.23%) and pneumonia (46.24%), when PM_2.5_ levels exceeded the proposed daily limit of 25 μg/m^3^, although not statistically significant for asthma and pneumonia.

### 4.1. PM_2.5_ Concentrations

PM_2.5_ levels in the city of Volos exceeded the proposed daily limit set by the air quality guidelines issued by WHO for 48.6% of the study period. The proposed mean annual limit was also exceeded. The highest PM_2.5_ levels were noted during winter and spring. Our findings are in accordance with a previous study set in the city of Volos by Moustris et al. [14]. The researchers showcased that, during a five-year period, the concentration of fine particulate matter in the city of Volos regularly exceeded the safety limit proposed by WHO, with the highest levels of air pollution being noted during the cold months (October–April).

The same trend has been noted in several urban centers worldwide [6,20], with 98% of Beijing’s population being exposed to harmful annual levels of PM_2.5_ pollution [6]. In addition to Beijing, a considerable number of other Asian developing metropolises, primarily including urban areas in China and India, are regularly exposed to high daily levels of fine particulate matter [21]. In Europe, air pollution has been shown to constitute a health hazard in several industrial Mediterranean cities [22], including Greek urban centers like Athens and Thessaloniki [23,24]. Therefore, while fine particulate matter pollution is primarily affecting huge metropolises, smaller urban areas are also gravely impacted.

Since fine particulate matter pollution is mainly anthropogenic in nature, it is no wonder that urban centers are more severely affected [25]. More specifically, the combustion of fuel by vehicles and power plants, as well as several industrial processes, are some of the main sources of fine particulate matter production. In the city of Volos, vehicular traffic, especially tourist traffic towards the seaport and tourist attractions, as well as the small industrial areas and cement industry, have been identified as the main sources of air pollution [14]. 

Financial crises have also been shown to lead to increased emissions of pathogenic air pollutants, especially PM_2.5_ [26]. This may be partially attributed to an increase of wood-burning in fireplaces and woodstoves [27], which has largely replaced heating oil in Greece, due to its lower price since the start of the economic crisis in 2010 [28].

In order to mitigate the effects of fine particulate matter on human health, environmental monitoring and evaluation, as well as strict environmental law enforcement, are necessary [29]. In an attempt to reduce PM_2.5_ levels, proper measures need to be taken, enabling the reduction of coal-based fuel usage and industrial fine particulate matter generation [30]. By implementing such measures, fine particulate matter levels in Beijing fell by 35% in a period of 20 years [31]. The strict environmental laws enforced in Beijing, as well as the Air Quality Guidelines by WHO can be utilized to minimize PM_2.5_ emissions in urban centers, in order to ameliorate the harmful effects of air pollution on human health.

### 4.2. Health Impacts of PM_2.5_

It has been previously established that high levels of PM_2.5_ can have various detrimental health effects [8]. The small aerodynamic diameter of PM_2.5_ allows the particles to reach the small alveoli [32]. Subsequently, PM_2.5_ may irritate or damage the alveolar wall and instigate lung dysfunction [33]. As a result, increased levels of PM_2.5_ pollution have been associated with a variety of acute and chronic respiratory diseases [10]. Moreover, fine particulate matter exposure has been associated with an increased incidence of respiratory diseases, such as upper and lower respiratory tract infections and asthma exacerbations, in pediatric patients [12,34]. 

Our results corroborate the above findings since the number of pediatric ED visits in the city of Volos increased as much as 32.44% in the days that PM_2.5_ levels raised higher than the proposed limit. The association between the short-term increase in PM_2.5_ levels and the incidence of several acute diseases has been previously established in both adult and pediatric patients, as ED visits, especially for respiratory causes, have been shown to significantly increase for three days following excess fine particulate matter pollution [35,36]. In a meta-analysis of 35 studies by Liu et al., the risk of respiratory tract diseases was significantly higher in pediatric patients [37]. Moreover, based on our analysis, the total number of ED visits in our setting can be calculated using the number of daily PM_2.5_ levels, utilizing the equation “Total number of ED visits = 8.546 + 0.104 × PM_2.5_ levels”.

The harmful effect of fine particulate matter on the respiratory system seems to be multifactorial and possibly includes immune response modification and inflammation [38] and reduced antimicrobial activity in the respiratory system [39]. It has been hypothesized that children are more susceptible to the effects of PM_2.5_, owing to their immature immune system and narrower airways [40]. Indeed, there have been reports that short-term exposure to fine particulate matter pollution increases the risk of acute upper and lower respiratory infections [41,42]. Similarly, in our study pediatric ED visits for URI increased by 32% when PM_2.5_ levels exceeded the daily proposed limit, suggesting that air pollution severely affects the risk of developing respiratory infections in children.

There is growing evidence that fine particulate matter pollution is a major instigator of asthma exacerbations [43,44]. Airborne pollutants seem to act as allergens, enhancing atopic sensitization and, thus, leading to asthma exacerbations and increased hospitalizations in children [45]. In our study, high PM_2.5_ levels were associated with an increase in pediatric ED visits for asthma-like symptoms, reaching an increase of 19.23%, although not statistically significant. These findings are consistent with the existing literature, showcasing that children are more likely than adults to exhibit asthma-like symptoms during periods of short-term exposure to high PM_2.5_ concentrations and are at an increased risk for hospitalization [44,46].

While the effect of fine particulate matter exposure to the development of respiratory symptoms has already been established, environmental health awareness and preparedness is not common amongst physicians [47]. For this reason, WHO published a report summarizing the harmful effects of air pollution on pediatric health. In this report, physicians are encouraged to update their knowledge on environmental health issues and inform parents about the dangers of exposure to urban pollution [48], proposing simple measures that can be taken in order to minimize exposure to high levels of fine particulate matter. These include indoor recreational activities, indoor air filters and efforts to reduce outdoor air infiltration, especially during periods in which PM_2.5_ levels exceed the proposed safety limit [49]. 

Individuals can be informed on the daily levels of air pollution through specially created websites, similar to the GreenYourAir website in Volos (http://greenyourair.org/link (accessed on 9 June 2021)) that show real-time measurements of PM_2.5_ levels, in order to limit their activities accordingly. It is also crucial for physicians to be vigilant during periods of high levels of fine particulate matter pollution and accurately inform and monitor their patients, especially those who are highly susceptible to the harmful effects of air pollution, like children suffering from asthma [48]. Furthermore, primary care centers and hospitals could hire environmental health specialists, in order to effectively manage the higher influx of patients who may present to the emergency department during periods of excess air pollution. They can work alongside physicians to appropriately educate them regarding the management of pollution-related health problems and raise environmental health awareness. Moreover, hospitals could be re-organized in order to allocate more staff and resources in EDs and primary care centers during periods of high PM_2.5_ pollution, in order to effectively optimize patient care. As previously mentioned, it is possible to use mathematical equations in order to estimate the number of ED visits based on the PM_2.5_ levels of the day. Using those equations, emergency physicians could anticipate the greater influx of patients which may present in the ED during days of high atmospheric pollution, in order to efficiently organize the ED personnel and resources, thus greatly improving patient management. However, it is important to highlight that the number of ED visits is also affected by several other reasons, such as season and viral outbreaks. While environmental medicine can be utilized in order to optimize ED patient care, a variety of factors have to be taken into consideration when managing ED resources and personnel.

### 4.3. Limitations

Our study has certain limitations that should be acknowledged. Firstly, our analysis implemented the mean values of the measured PM_2.5_ levels, thus not accurately reflecting the individual exposure of our study participants. Furthermore, as the diagnoses were performed in an emergency setting, it is possible that some of them were inaccurate, leading to misclassification. 

## 5. Conclusions

The mean annual concentration of PM_2.5_ in the city of Volos was 30.03 μg/m^3^, much higher than the WHO yearly limit of 10 μg/m^3^. When PM_2.5_ levels were above the daily limit, an increase of 32.44% was observed in daily pediatric ED visits for respiratory-related conditions, such as asthma, pneumonia and URI, with the increase being higher during winter and spring. 

Local data from different cities-environments across Greece will improve our understanding of air pollution on the health of children and will aid policymakers in decisions that relate to the sustainability of development and prevention of air pollution. A global prevention policy should be designed in order to combat anthropogenic air pollution as a complement to the correct handling of the adverse health effects associated with air pollution. International cooperation in terms of research, development, administration policy, monitoring, and politics is vital for effective pollution control. These challenges need to be addressed if we are to protect the health of children in the coming generations.

## Figures and Tables

**Table 1:** Table presenting the number of pediatric ED visits in relation to daily PM_2.5_ levels.

Population	PM_2.5_ Level	Total Days	Number of ED Visits	Mean ED Visits/Day (SD)	% Increase in Mean ED Visits	*p*-Value
All patients	-	366	4271	11.67 (5.665)	-	-
All patients	<25 μg/m^3^	188	1895	10.08 (4.087)	32.44	<0.001
	≥25 μg/m^3^	178	2376	13.35 (6.564)		
URI	<25 μg/m^3^	188	1744	9.28 (3.88)	32	<0.001
	≥25 μg/m^3^	178	2180	12.25 (6.216)		
Asthma	<25 μg/m^3^	188	97	0.52 (0.817)	19.23	0.23
	≥25 μg/m^3^	178	111	0.62 (0.876)		
Pneumonia	<25 μg/m^3^	188	39	0.21 (0.468)	42.86	0.08
	≥25 μg/m^3^	178	54	0.30 (0.561)		

**Table 2:** Pediatric ED visits according to PM_2.5_ levels in each season.

Season	PM_2.5_ Level	Total Days	Number of ED Visits	Mean ED Visits/Day (SD)	% Increase in Mean ED Visits	*p*-Value
Winter	<25 μg/m^3^	18	265	14.72 (4.663)	14.20	0.271
	≥25 μg/m^3^	73	1227	16.81 (7.626)		
Spring	<25 μg/m^3^	42	424	10.10 (3.58)	21.19	0.018
	≥25 μg/m^3^	50	612	12.24 (4.728)		
Summer	<25 μg/m^3^	74	695	9.39 (3.572)	6.50	0.520
	≥25 μg/m^3^	18	180	10.00 (3.614)		
Autumn	<25 μg/m^3^	54	511	9.46 (3.98)	2	0.822
	≥25 μg/m^3^	37	357	9.65 (3.646)		

**Table 3:** ED visits for URI according to season and PM_2.5_ levels.

Season	PM_2.5_ Level	Total Days	Number of ED Visits	Mean ED Visits/Day (SD)	% Increase in Mean ED Visits	*p*-Value
Winter	<25 μg/m^3^	18	242	13.44 (4.592)	13.84	0.302
	≥25 μg/m^3^	73	1117	15.30 (7.222)		
Spring	<25 μg/m^3^	42	385	9.17 (3.435)	23.66	0.016
	≥25 μg/m^3^	50	567	11.34 (4.792)		
Summer	<25 μg/m^3^	74	656	8.86 (3.410)	7.22	0.479
	≥25 μg/m^3^	18	171	9.50 (3.365)		
Autumn	<25 μg/m^3^	54	461	8.54 (3.81)	2.81	0.757
	≥25 μg/m^3^	37	325	8.78 (3.591)		

## Data Availability

The data that support the findings of this study are available from the corresponding author (M.M.), upon reasonable request.

## Supplementary Materials

The following are available online at https://www.mdpi.com/article/10.3390/ijerph18126384/s1, Supplementary Table S1: Table presenting the total number of ED visits during the study period according to patient age.
