# Supplementary material for: Effect of PM2.5 Levels on Respiratory Pediatric ED Visits in a Semi-Urban Greek Peninsula
Source: Int J Environ Res Public Health. 2021 Jun 12;18(12):6384. doi: 10.3390/ijerph18126384 (PMC8296213; doi:10.3390/ijerph18126384)
Supplement: Supplementary file 1 [file ijerph-18-06384-s001.zip › ijerph-1233305-supplementary.pdf]

Age	Number of ED Visits
0-1	677
2-5	1579
6-12	1493
13-15	466
15-18	56

Supplementary Table S1. Table presenting the total number of ED visits during the study period according to patient age.
